# Impact of clozapine monotherapy on gut microbiota and metabolism in people with schizophrenia

**DOI:** 10.3389/fmicb.2023.1253156

**Published:** 2023-09-06

**Authors:** Feiyan Yin, Zhidao Shi, Xiquan Ma, Kai Ding, Yuan Zhang, Sha Ma

**Affiliations:** ^1^Clinical Research Center for Mental Disorders, Shanghai Pudong New Area Mental Health Center, School of Medicine, Tongji University, Shanghai, China; ^2^Department of Developmental and Behavioral Pediatrics, Shanghai Children’s Medical Center, School of Medicine, Shanghai Jiao Tong University, Shanghai, China; ^3^State Key Laboratory of Subtropical Silviculture, School of Forestry and Biotechnology, Zhejiang A&F University, Hangzhou, China

**Keywords:** metabolic syndrome, gut microbiota, schizophrenia, clozapine, monotherapy

## Abstract

**Background:**

Clozapine is considered one of the most effective antipsychotic drugs, but it is most likely to cause metabolic abnormalities. Researchers have studied the causes of metabolic abnormalities caused by clozapine from multiple perspectives, but the reasons remain unclear.

**Purpose:**

Characterize the gut microbiota of people with schizophrenia taking clozapine, exploring the association between gut microbiota and glucose lipid metabolic markers in schizophrenia patients taking clozapine.

**Research design:**

Sixty-one long-term inpatients with schizophrenia in clozapine monotherapy were selected as study subjects. We got four subgroups by sex and the presence of metabolic syndrome.

**Data analysis:**

16s analysis technology was applied at the genus level to determine the classification of gut microbiota. Then we compared the characteristics of gut microbiota and the association of gut microbiota with glucose lipid metabolic markers in each group.

**Findings:**

We found differences in the diversity of gut microbiota among groups. The association between gut microbiota and glucose lipid metabolic markers was complicated. Gender was an important differentiating factor. *Oscillibacter* has a low abundance. However, it was the only genus associated with glycemic or lipids in each group. Among metabolic syndromes, *Gemmiger* was positively correlated with most lipids in females but negatively correlated in males, showing gender differences. In female non-metabolic syndromes, *Bifidobacterium* lost its probiotic character; instead, showing pathogenicity, which has strong positive correlations with fasting blood glucose and low-density lipoprotein but negative correlations with Apolipoprotein A1. Maybe schizophrenia, taking clozapine, and gender factors influenced the gut microbiota, which complicated our findings. The significance of the results remains to be determined by in-depth studies.

## 1. Introduction

Second-generation antipsychotics are currently the mainstay of treatment for schizophrenia. Clozapine is considered the prototype and representative of second-generation antipsychotics (Stahl, 2013). Clozapine is significantly better than other antipsychotics in reducing psychotic symptoms, violence, and persistent aggression in patients with schizophrenia (Frogley et al., 2012). It is the only antipsychotic used as the standard of care for refractory schizophrenia in the treatment guidelines for schizophrenia developed in several countries (Stahl et al., 2013; Nice, 2014; Warnez and Alessi-Severini, 2014). The U.S. Food and Drug Administration (FDA) approved clozapine as the only drug to reduce suicidal behavior in patients with schizophrenia (Versacloz, 1989).

Patients on clozapine have the most significant increases in cholesterol, triglycerides, and blood glucose (Krakowski et al., 2009), which limits its use in clinical practice (Citrome et al., 2016). Up to 60% of people with schizophrenia taking clozapine develop metabolic disorders such as dyslipidemia, insulin resistance, and type 2 diabetes (Brunero et al., 2009; Meltzer, 2012; Mitchell et al., 2013). Some studies have suggested that the metabolic disorders in patients are related to neurotransmitters such as serotonin 5-HT2A/2C receptors and histamine H1 receptors (Kim et al., 2007; Esen-Danacı et al., 2008); others have suggested that it may be the result of clozapine affecting the production or the secretion mechanism of adipocytokines such as adiponectin, leptin, and resistin in the body (Yuen et al., 2021). Some other studies believe that clozapine induces the production of inflammatory cytokines such as interleukins and tumor necrosis factors in the body. The monocyte infiltration and inflammatory state lead to insulin resistance, subsequently interfering with the insulin’s antilipolytic effects, thus causing metabolic disorders (Leonard et al., 2012). Several studies have found that variants in certain genes (especially LEP, HTR2C, and SREBF2) may make patients taking clozapine genetically susceptible to metabolic disorders (Suetani et al., 2017). Overall, the current studies represent a variety of cellular, biochemical, molecular, and physiological pathways; The findings are complex and even contradictory. The mechanisms underlying clozapine-induced metabolic disorders are not well understood to date, which may result from a combination of factors; there may also be some mechanisms that have not yet been identified (Panariello et al., 2011).

More and more evidence shows that the gut microbiota is important in the body’s energy metabolism (Fan and Pedersen, 2021). Influenced by specific factors, changes in the abundance and composition of the gut microbiota associated with metabolic abnormalities may lead to metabolic disorders (Singh et al., 2015; DeGruttola et al., 2016). Type 2 diabetes were characterized by a moderate degree of gut microbial dysbiosis (Qin et al., 2012). A strong association exists between certain gut microbiota composition, body mass index (BMI) changes, and blood lipid levels (Fu et al., 2015). Specific gut microbiota may restrain the expression of fasting-induced adipose factor, promote cellular uptake of fatty acids and enhance the accumulation of triglycerides in adipose tissue (Backhed et al., 2007). Some gut microbiota also produces short-chain fatty acids (SCFA) via anaerobic fermentation of dietary fiber, which in turn affects cholesterol and fat synthesis through the gut microbiota-liver axis pathway (Singh et al., 2015).

Due to limitations in research methods, the important role of gut microbiota has not been considered in previous studies investigating the mechanisms underlying metabolic abnormalities in patients with schizophrenia induced by second-generation antipsychotics. In fact, with the application of high-throughput omics technologies in the field of gut microbiota, the comprehensive study of the structure and function of all microbial species in the gut microbiota has only been achieved. Studies have found that some gut microbiota were associated with the severity of symptoms (Li et al., 2020). Risperidone treatment induces significant changes in some gut microbiota (Bahr et al., 2015a,b; Yuan et al., 2018). The changes in gut microbiota composition may reflect the effects of metabolic disorders following risperidone treatment (Xue et al., 2021). Risperidone leads to significant changes in the gut microbiota, which are mechanistically related to weight gain by suppressing energy expenditure (Bahr et al., 2015b). The gut microbiota also has a role in the cycle of metabolic abnormality associated with olanzapine. Olanzapine has contributed to a shift in gut microbiota toward an “obesogenic” bacterial profile (Morgan et al., 2014); increased the abundance of Short Chain Fatty Acids (SCFA) producing gut microbiota increased the levels of SCFAs, which are strongly associated with the development of lipid accumulation (Chang et al., 2023). Chronic olanzapine treatment altered the gut microbiota of rats and caused severe metabolic side effects in rats. After killing the gut microbiota in rats with a combined antibiotic cocktail, indicators of metabolic disorders such as blood lipids, adipose tissue inflammation, and fat deposition were significantly improved (Davey et al., 2013). The abnormal lipid metabolism induced by olanzapine may be strongly associated with the vagus-mediated gut microbiota-brain axis (Zhu et al., 2022).

Although certain second-generation antipsychotics alter the abundance and composition of the gut microbiota and induce metabolic disturbances in people with schizophrenia (Chen et al., 2023), it is unclear whether gut microbiota is also involved in the mechanism of metabolic abnormalities caused by clozapine. Therefore, We analyzed the characterization of gut microbiota in people with schizophrenia with and without metabolic syndrome (MetS) after monotherapy with clozapine and the relationship between gut microbiota and Glycemic or lipids.

Despite its best efficacy among all antipsychotic drugs, its clinical application is restricted due to the greatest metabolic side effects. Therefore, further exploring the mechanisms of metabolic side effects induced by clozapine in people with schizophrenia would provide a theoretical basis for future targeted interventions.

## 2. Materials and methods

### 2.1. Ethics approval and consent to participate

The study was conducted according to the guidelines of the Declaration of Helsinki and approved by the Institutional Ethics Committee of Clinical Research Center for Mental Disorders, Shanghai Pudong New Area Mental Health Center, School of Medicine, Tongji University (protocol code: PDJWLL2019020 and date of approval: 5 July 2019). It was registered in the Chinese Clinical Trials Registry (Registration number: ChiCTR2300069814). All participants gave their informed consent for inclusion before participating in the study.

### 2.2. Study design and participants

The study was conducted among inpatients at the Pudong New Area Mental Health Center in Shanghai, China, and data were collected from October 2022 to April 2023. In order to minimize heterogeneity in the study population, long-term hospitalized patients with chronic schizophrenia were recruited into the study. The hospital restaurant supplies their daily food. They lived in the same environment for a long time, shared the same recipes as all inpatients, and had similar portion sizes. All this will minimize the differences in gut microbiota affected by different living environments and foods.

The participants recruited for this study were schizophrenia in clozapine monotherapy (SCM), with MetS (MA) and without MetS (MN). Study participants met the diagnostic criteria for schizophrenia in the International Classification of Diseases, Tenth Revision (ICD-10), were Han Chinese, aged >18 years, currently on single-agent oral clozapine therapy for more than six consecutive months, and volunteered to participate. Participants in the MA group met the diagnostic criteria for metabolic syndrome of the Chinese Diabetes Association (2021) and Chen et al. (2023). In contrast, participants in MN did not meet the diagnostic criteria for metabolic syndrome. Participants were excluded from this study if they (1) smoked; (2) had a history of bowel cancer; (3) had a history of inflammation; (4) had used antibiotics in the last 6 months, or (5) had received invasive medical interventions during the previous 6 months.

### 2.3. Clinical study parameters

We collected data on age, gender, blood pressure (BP), body mass index (BMI), and waist circumference (WC) of participants. We collected blood samples from participants under fasting conditions. Venipuncture was performed using vacuum blood collection tubes, and blood was stored in blood collection tubes, serum separator tubes, and EDTA Vacutainer (Becton Dickinson, Franklin Lakes, NJ, USA). Blood samples were centrifuged at 1,500 × *g* for 15 min at 4°C. Standard laboratory methods and certified biochemical and hematological tests were performed using an automated analyzer (Roche Diagnostics, Mannheim, Germany).

Participants’ fasting blood glucose (FBG), Glycated albumin (GA), triglycerides (TG), total cholesterol (TC), High-density lipoprotein cholesterol (HDL-C), low-density lipoprotein cholesterol (LDL-C), Small dense low-density lipoprotein-cholesterol (sdLDL-C), Apolipoprotein A1 (ApoA1), Apolipoprotein B (ApoB), Apolipoprotein E (ApoE) and lipoprotein (a) [Lp (a)].

### 2.4. DNA extraction, PCR amplification, and 16S rRNA gene sequencing

Deoxyribonucleic acid was extracted from each fecal sample following the manufacturer’s protocols of the QIAamp Fast DNA Stool Mini Kit (Qiagen, Germany). The V3-V4 region of the bacteria 16S ribosomal RNA genes was amplified by PCR (95°C for 3 min, followed by 30 cycles at 98°C for 20 s, 58°C for 15 s, and 72°C for 40 s and a final extension at 72°C for 5 min) using barcoded primers 341F (5′-CCTACGGGRSGCAGCAG-3′) and 806R (5′-GGACTACVVGGGTATCTAATC-3′). PCR reactions were performed in a 25 μL mixture containing 12.5 μL of KFX HiFi 2 × PCR Master Mix, 1 μL of each primer (10 μM), 50 ng of template DNA and dd H2O. Amplicons were purified using the AxyPrep DNA Gel Extraction Kit (Axygen Biosciences, Union City, CA, US) and quantified using Qubit^®^2.0 (Invitrogen, US). All quantified amplicons were pooled to equalize concentrations for sequencing using Illumina NovaSeq (Illumina, Inc., San Diego, CA, USA). The total samples resulted in 2,181,677 tags, averaging 35,765 tags per sample. DNA extraction, library construction, and sequencing were conducted at Realbio Genomics Institute (Shanghai, China).

### 2.5. Taxonomy classification and statistical analysis

High-quality 16S tags were clustered into Operational Taxonomic Units (OTUs) with 97% similarity using Usearch (version 7.0.1090) in QIIME (version 1.9.1). Taxonomy was assigned using the RDP classifier (https://sourceforge.net/projects/rdp-classifier/) using a confidence threshold of 0.8. Student’s unpaired *t*-test and one-way analysis of variance (ANOVA) was performed using SPSS Statistics software (V22.0, IBM, Armonk, NY, USA), and a *P*-value of < 0.05 was considered statistically significant. Duncan’s test was performed to compare differences in gut bacterial diversity among different subgroups of schizophrenia patients receiving clozapine. Changes in the structure of gut bacterial communities were analyzed by beta-diversity based on Bray-Curtis distances and visualized by principal coordinate analysis (PCoA) of the “vegan” package. The significance of differences in composition between bacterial communities was calculated using the permutational multivariate analysis of variance (PERMANOVA). STAMP software was used to analyze differences in the composition of the gut microbiota community at the phylum and genus levels. Redundancy analysis was used to analyze the relationship between gut bacterial community structure and Clinical index.

## 3. Results

### 3.1. Baseline characteristics

A total of 61 SCM were recruited, including 28 in the MA group (16 females and 12 males) and 33 in the MN group (14 females and 19 males). The two groups were comparable in age, gender, duration of clozapine monotherapy (DCM), and Clozapine dosing (CD). The FBG values were higher in the MA group than in the MN group (*p* < 0.05). The proportion of WC, BP, GA, TG, sdLDL-C, and ApoB was significantly higher in the MA group than in the MN group (*p* < 0.01) (Table 1).

**TABLE 1:** Demographic and blood test data of MA and MN groups.

Variables	MA	MN	*P*-value
N	28	33	
GC (Female/male)	16/12	14/19	0.252
Age (year)	57.71 ± 11.04	55.27 ± 13.36	0.445
DCM (mouth)	14.39 ± 6.51	16.24 ± 10.21	0.412
CD (mg)	200.89 ± 75.61	244.70 ± 99.36	0.061
MBMI (kg/m2)	24.21 ± 2.26	23.20 ± 4.17	0.236
WC	96.43 ± 6.94	89.42 ± 12.22	**0.007**
BP	24	4	**<0.001**
FBG (mmol/L)	5.76 ± 1.11	5.27 ± 0.36	**0.032**
GA	6.74 ± 0.97	5.31 ± 0.81	**<0.001**
TC (mmol/L)	4.29 ± 0.72	4.19 ± 0.55	0.534
TG (mmol/L)	2.01 ± 0.89	1.04 ± 0.33	**<0.001**
HDL-C	1.09 ± 0.27	1.19 ± 0.25	0.116
LDL-C	2.58 ± 0.56	2.45 ± 0.44	0.311
sdLDL-C	317.60 ± 126.39	236.52 ± 66.05	**<0.001**
Lp (a)	234.36 ± 248.24	272.42 ± 181.58	0.493
Apo-AI	1.23 ± 0.18	1.22 ± 0.14	0.898
Apo-B	0.72 ± 0.13	0.63 ± 0.07	**0.003**
Apo-E	3.69 ± 0.72	3.39 ± 0.59	0.079

MA, schizophrenia in clozapine monotherapy who was diagnosed with metabolic syndrome; MN, schizophrenia in clozapine monotherapy who did not develop the metabolic syndrome; N, the number of samples per group; GC, Gender composition; DCM, duration of clozapine monotherapy; CD, Clozapine dosing; MBMI, Mean value of body mass index, WC, waist circumference; BP, Number of people with high blood pressure; FBG, fasting blood glucose; GA, glycated albumin; TC, total cholesterol; TG, triglycerides; HDL-C, High density lipoprotein cholesterol; LDL-C, low-density lipoprotein cholesterol; sdLDL-C, Small dense low-density lipoprotein-cholesterol; Lp (a), lipoprotein (a), ApoA1, Apolipoprotein A1; ApoB, Apolipoprotein B; ApoE, Apolipoprotein E. Bold values indicate significance at the level of *P* < 0.05.

Female participants in the MA group (AF) had significantly higher WC, HDL-C, and Apo-AI than male participants in the MA group (AM) (*P* < 0.01). At the same time, sdLDL-C was higher in the AF than in the AM (*P* < 0.05). Female participants in the MN group (NF) had higher WC, TC, HDL-C, Apo-AI, and Apo-E than male participants in the MN group (NM) (*P* < 0.05). GA was significantly higher in the NM group than in the NF group (*p* < 0.01) (Table 2).

**TABLE 2:** Demographic and blood test data of four subgroups.

Variables	MA			MN		
	**AF**	**AM**	***P*-value**	**NF**	**NM**	***P*-value**
**N**	16	12		14	19	
Age (year)	55.88 ± 13.42	60.17 ± 6.52	0.318	50.57 ± 11.40	58.74 ± 13.91	0.082
DCM (mouth)	13.88 ± 5.95	15.08 ±	7.39	0.636	14.86 ± 0.21	3.53 ± 6.08
CD (mg)	215.63 ± 72.38	181.25 ± 78.43	0.241	246.43	105.55	243.42 ± 97.48
MBMI (kg/m2)	24.56 ± 2.48	23.75 ± 1.93	0.356	24.11 ± 5.04	22.54 ± 3.38	0.323
WC	99.94 ± 5.36	91.75 ± 6.11	**<0.001**	94.86 ± 12.22	85.42 ± 10.84	**0.026**
BP	15	9	0.168	1	3	0.459
FBG (mmol/L)	5.92 ± 1.13	5.55 ± 1.09	0.391	5.26 ± 0.32	5.28 ± 0.40	0.865
GA	6.72 ± 1.01	6.77 ± 0.96	0.893	4.79 ± 0.72	5.69 ± 0.66	**<0.001**
TC (mmol/L)	4.31 ± 0.77	4.27 ± 0.67	0.883	4.44 ± 0.44	4.00 ± 0.56	**0.021**
TG (mmol/L)	1.81 ± 0.68	2.27 ± 1.09	0.177	1.13 ± 0.40	0.98 ± 0.25	0.221
TyG	8.97 ± 0.32	9.10 ± 0.31	0.287	8.40 ± 0.37	8.29 ± 0.29	0.331
HDL-C	1.20 ± 0.28	0.93 ± 0.18	**0.006**	1.30 ± 0.26	1.11 ± 0.21	**0.032**
LDL-C	2.47 ± 0.55	2.73 ± 0.56	0.213	2.55 ± 0.39	2.38 ± 0.48	0.272
sdLDL-C	274.9 ± 116.31	374.46 ± 120.79	**0.037**	246.51 ± 80.66	229.17 ± 54.05	0.465
Lp (a)	196.25 ± 159.15	285.18 ± 334.35	0.408	307.64 ± 206.52	246.47 ± 161.67	0.347
Apo-AI	1.30 ± 0.17	1.12 ± 0.15	**0.008**	1.28 ± 0.16	1.17 ± 0.09	**0.036**
Apo-B	0.70 ± 0.14	0.74 ± 0.12	0.422	0.66 ± 0.07	0.61 ± 0.07	0.094

AF, female participants in the MA group; AM, male participants in the MA group; NF, female participants in the MN group; NM, male participants in the MN group. Bold values indicate significance at the level of *P* < 0.05.

### 3.2. Gut microbiota status in study participants

#### 3.2.1. Diversity of gut microbiota in each group

**FIGURE 1:**
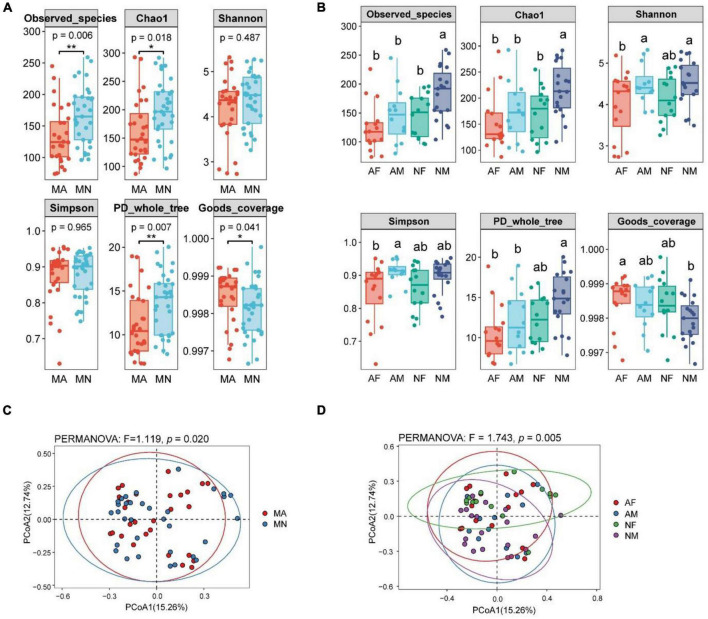
Differences in α-diversity and β-diversity of gut microbiota of participants in different groups. The lowercase letters represented significant differences at the 0.05 level. MA, schizophrenia in clozapine monotherapy who was diagnosed with metabolic syndrome; MN, schizophrenia in clozapine monotherapy who did not develop the metabolic syndrome; AF, female participants in MA; AM, male participants in MA; NF, female participants in the MN; NM, male participants in MN; **(A)**, Differences in α-diversity between MA and MN groups; **(B)**, Differences in α-diversity among AF, AM, NF, and NM groups; **(C)**, Differences in β-diversity be-tween MA and MN groups; **(D)**, Differences in β-diversity among AF, AM, NF, and NM groups. *P < 0.05 and ***P < 0.01.

There were significant differences in the α-diversity of gut microbiota In the MA and MN groups. Observed_species, Chao1, and PD_whole_tree indices of gut microbiota were significantly higher in the MN group than in the MA group. In contrast, the opposite was true for the Goods_coverage index (Figure 1A). Within the MA or MN groups, the diversity of gut microbiota was higher in male participants than in female participants, i.e., the diversity of AM group was higher than that of the AF group, and NM was higher than that of the NF. In addition, there were no significant differences in the α-diversity of gut microbiota between the AF and NF groups. However, the α-diversity of gut microbiota in the NM group was significantly higher than that in the AM group (Figure 1B). For β-diversity, principal coordinates analysis (PCoA) of Bray-Curtis distances showed that the community composition of gut microbiota was significantly different between MA and MN (PERMANOVA, *F* = 1.119, *P* < 0.05) (Figure 1C). In addition, there were significant differences in gut microbiota community composition between the four subgroups (PERMANOVA, *F* = 1.743, *P* < 0.01) (Figure 1D).

#### 3.2.2. Composition of gut microbiota among different groups

**FIGURE 2:**
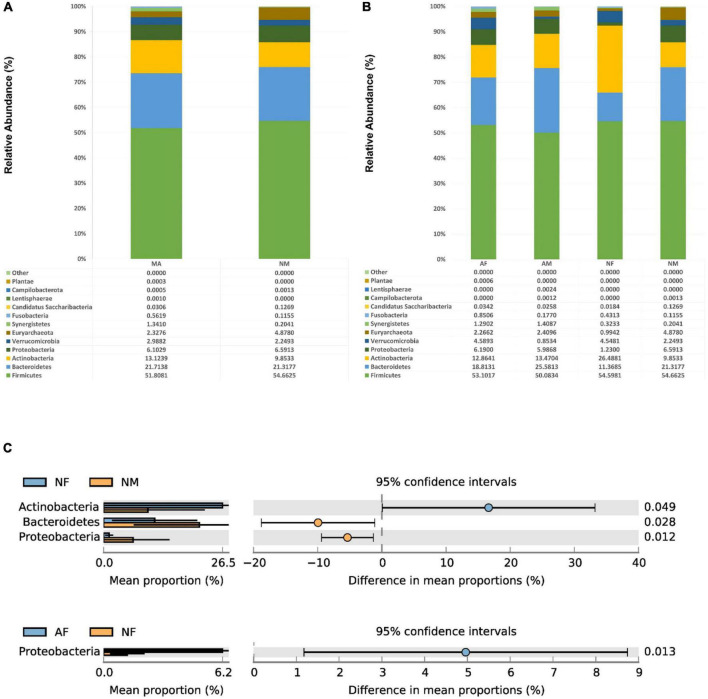
At the phylum level, the composition and the differences of gut microbiota in each group. **(A)**, Composition of the gut microbiota in the MA and MN groups at the phylum level; **(B)**, Composition of the gut microbiota in the AF, AM, NF, and NM groups at the phylum level; **(C)**, At the phylum level, statistically significant groups comparisons are shown.

At the phylum level, The Firmicutes, Bacteroidetes, Actinobacteria, and Proteo-bacteria were the most abundant four phyla in MA, MN, AF, AM, and NM groups. Moreover, the NF group’s most abundant four phyla were Firmicutes, Actinobacteria, Bacteroidetes, and Verrucomicrobia, respectively (Figures 2A, B). No significant differences were found in the abundance of each phylum in both MA and MN groups. The abundance of proteobacteria in the NF group was significantly lower than that in the NM and AF groups. The abundance of Bacteroidetes in the NM group was higher than that in the NF group. The abundance of Actinobacteria in the NM group was lower than that in the NF group (Figure 2C).

**FIGURE 3:**
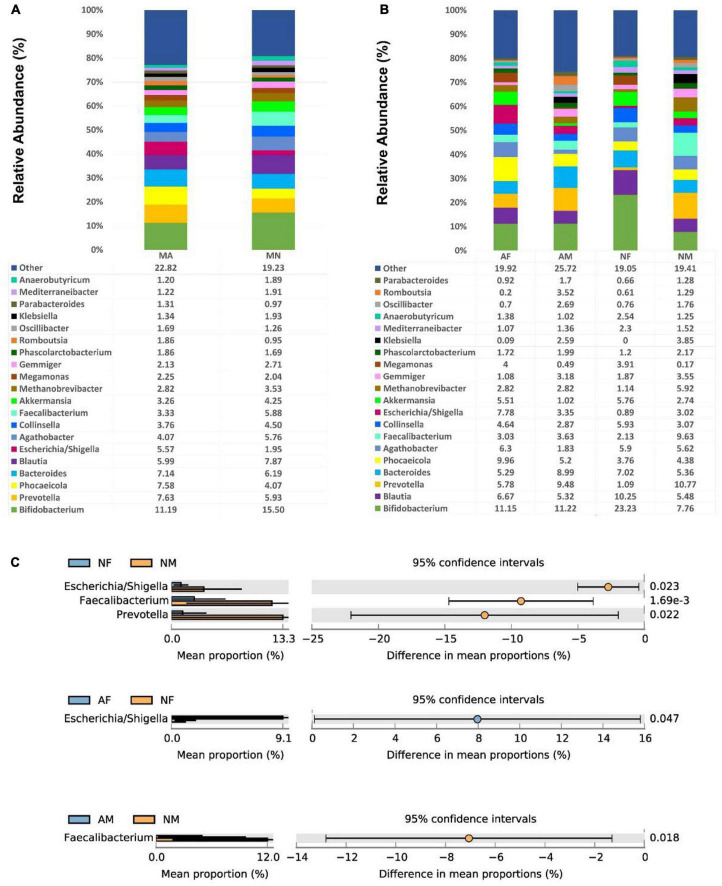
At the genus level, the composition and the differences of gut microbiota in each group. **(A)**, Composition of the gut microbiota in the MA and MN groups at the genus level; **(B)**, Composition of the gut microbiota in the AF, AM, NF, and NM groups at the genus level; **(C)**, At the genus level, statistically significant groups comparisons are shown.

At the genus level, There were no significant differences among the genera in both MA and MN groups. *Bifidobacterium* was the most abundant in all four sub-groups, with a relative abundance of 23.23% in the NF group. In addition, *Blautia*, *Bacteroides*, *Prevotella*, *Phocaeicola*, *Agathobacter*, and *Collinsella* were also relatively abundant in each group (Figures 3A, B). The abundance of *Faecalibacterium* in the NM group (9.63%) was statistically different from that in the AM (3.63%) and NF (2.13%) groups (*P* < 0.01). The *Escherichia*/*Shigella* and *Prevotella* were also significantly more abundant in the NM group than in the NF group.

Among all female participants, there was a significantly higher abundance of *Escherichia*/*Shigella* in the AF group (7.78%) than in the NF group (0.89%) (*P* < 0.05). In contrast, among all male participants, there was a significantly higher abundance of *Faecalibacterium* in the NM group (9.63%) than in the AM group (3.63%) (*P* < 0.05) (Figure 3C).

#### 3.2.3. Correlation analysis between gut microbiota abundance and clinical parameters

**FIGURE 4:**
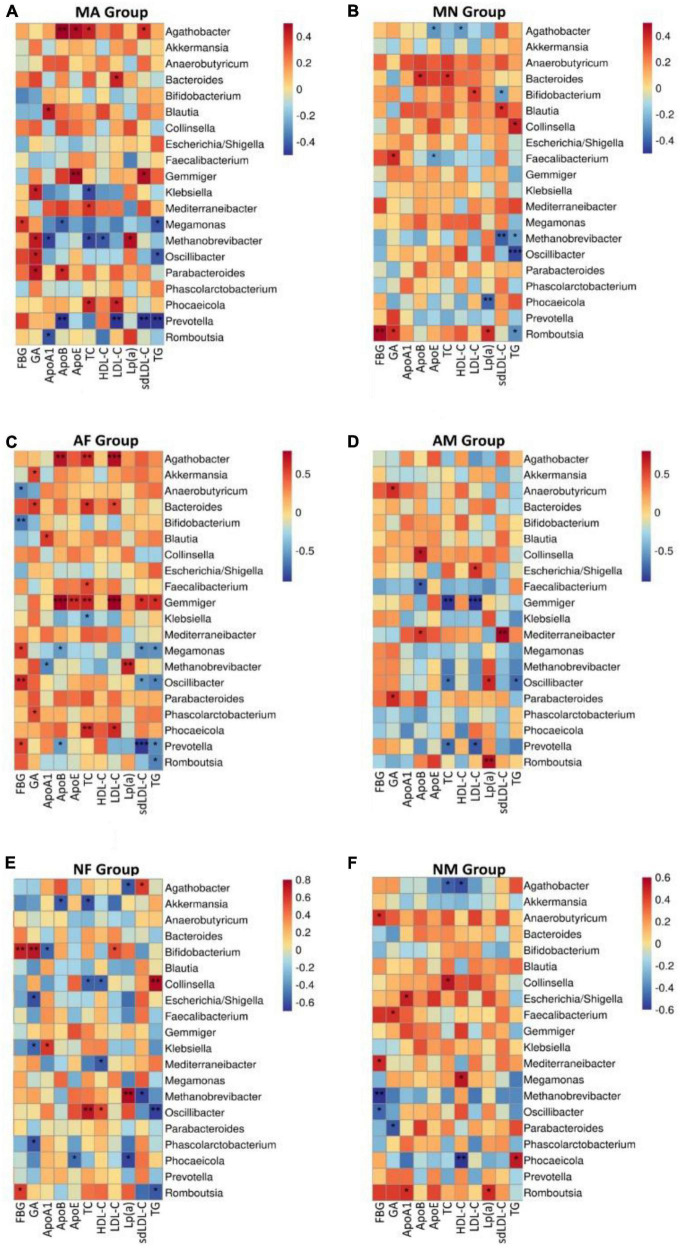
At the genus level, correlation analysis of the abundance of gut microbiota with glycemic or lipids in each group. **(A)**, Correlation analysis of the abundance of gut microbiota with glycemic or lipids in MA group at the genus level; **(B)**, Correlation analysis of the abundance of gut microbiota with glycemic or lipids in MN group at the genus level; **(C)**, Correlation analysis of the abundance of gut microbiota with glycemic or lipids in AF group at the genus level; **(D)**, Correlation analysis of the abundance of gut microbiota with glycemic or lipids in AM group at the genus level; **(E)**, Correlation analysis of the abundance of gut microbiota with glycemic or lipids in NF group at the genus level; **(F)**, Correlation analysis of the abundance of gut microbiota with glycemic or lipids in NM group at the genus level. *P < 0.05 and **P < 0.01.

We performed a correlation analysis between the abundance of all gut microbial genera and glycemic or lipids in each group. Overall, the relationship between gut microbial genera and clinical indicators in MA and MN is quite different. Among all genera, only the abundance of *Oscillibacter* was positively correlated with TG of both MA and MN. *Megamonas* was positively associated with FBG in MA, but Romboutsia was positively associated with FBG in the MN group. In the MA group *Methanobrevibacter*, *Oscillibacter*, *Parabacteroides*, and *Klebsiella* were positively correlated with GA. In the MN group, it was *Romboutsia* and *Faecalibacterium* that were positively correlated with GA. *Agathobacter* showed a mostly positive correlation with the serum lipid profiles in the MA group. However, it showed a mostly negative correlation with the HDL-C and ApoE in the MN group (Figures 4A, B).

The association between the abundance of gut microbial genera and glycemic or lipids showed some gender differences also. We found that *Gemmiger* all showed positive correlations with serum lipid profiles except HDL-C and Lp (a) in the AF group (Figure 4C). In contrast, *Gemmiger* was predominantly negatively correlated with TC and LDL-C (Figure 4D). The association between gut microbiota and glycemic indicators also differed significantly in the AF and AM groups. *Prevotella*, *Oscillibacter*, and *Megamonas* were positively associated with FBG in the AF group, while *Anaerobutyricum* and *Bifidobacterium* were negatively associated with FBG. No genus was associated with FBG in the AM group, but *Parabacteroides* and *Anaerobutyricum* were positively associated with GA in the AM group. In the AF group, *Agathobacter* was positively correlated with TC, LHL-C, and Apo-B, while in the AM group, *Agathobacter* did not show any association with blood glucose indicators or serum lipid profiles (Figures 4C, D).

There was also a degree of difference between the NF and NM subgroups; for example, Phocaeicola was positively correlated with TG and negatively correlated with HDL-C in the NM group, while in the NF group, Phocaeicola was negatively correlated with Lp (a) and Apo-E. In the NF group, *Collinsella* was positively correlated with TG and negatively correlated with TC and HDL. In contrast, in the NM group, *Collinsella* was positively correlated with TC *Bifidobacterium* and was positively correlated with FBG and GA in the NF group, while not associated with them in the NM group (Figures 4E, F).

In addition, the association between their gut microbiota and glycemic or lipids was different when comparing the same-gender subgroups. In the two female groups, AF and NF, this difference was mainly shown in the association of *Gemmiger*, *Collinsella*, and *Agathobacter* with glycemic or lipids (Figures 4C, E). In the two male groups, AM and NM, this difference was mainly shown in the association of *Agathobacter*, *Phocaeicola*, *Prevotella*, and *Romboutsia* with glycemic or lipids (Figures 4D, F).

It is worth pointing out that, as a good lipoprotein (Qin et al., 2012), HDL-C was not associated with any of the genera in the AF and AM subgroups. In contrast, HDL-C was positively associated with *Oscillibacter* and negatively associated with *Mediterraneibacter* and *Collinsella* in the NF group. In the NM group, HDL-C was negatively correlated with Phocaeicola and *Agathobacter* and positively correlated with *Megamonas* (Figure 4).

Surprisingly, the *Oscillibacter* genus seems unaffected by the metabolic and gender grouping methods. It was negatively correlated with TG in all groups, which is noteworthy, although there were no statistical differences in the NM group.

## 4. Discussion

The mechanism of clozapine-induced metabolic abnormalities is unclear, and there might be other unknown mechanisms (Panariello et al., 2011). To date, relatively few studies have been conducted to analyze the gut microbiota of patients with antipsychotic-induced metabolic syndrome. To our knowledge, this is the first study to analyze the association between metabolic syndrome and gut microbiota in SCM.

Previous studies have confirmed that antipsychotics can induce changes in the diversity and composition of the gut microbiota (Chen et al., 2023). The present study also found that the α-diversity index was significantly lower in the MA group of SCM than that in the MN group of SCM, and there was also a significant difference in the β-diversity of the gut microbiota between the MA and MN groups of SCM.

Previous studies have not agreed on the gut microbiota characteristics of patients with metabolic syndrome in the general population (Armougom et al., 2009; Schwiertz et al., 2010). A He et al. (2018) survey of gut microbiota in patients with metabolic syndrome in the general population in Guangdong, China, found that Bacteroidetes, Proteobacteria, and Firmicutes ranked among the top three abundance of gut microbiota and account for 92.3% of the total abundance of gut microbiota in metabolic syndrome patients. In contrast, this study found that Firmicutes, Bacteroidetes, and Actinobacteria were ranked in the top three abundance in MA, MN, and the four subgroups classified by gender, which implies that the composition of the gut microbiota of SCM may differ from that of the general population.

There were no significant differences in the abundance of each phylum between the MA and MN groups in this study. In contrast, another study conducted in 2021 in Shanghai, China, analyzing the gut microbiota of males with schizophrenia with and without MetS, found that the abundance of Bacteroidetes, Proteobacteria, and Synergistetes was higher in the MetS group than in the non-MetS group; While the abundance of Lentisphaerae, Fusobacteria, and Firmicutes was higher in the non-MetS group (Xing et al., 2022).

This difference may be because the participants in that study were taking a wider variety of antipsychotics, unlike the present study, which included schizophrenia patients treated with clozapine monotherapy. Studies have found that taking different antipsychotic medications can have different effects on the composition and abundance of gut microbiota (Chen et al., 2023). We speculate that it is this reason that accounts for this discrepancy.

Due to the increased abundance of Proteobacteria in nutrition, metabolic disorders, and inflammation disorders, Proteobacteria is called the microbial signature of these diseases (Shin et al., 2015; Rizzatti et al., 2017). In this study, we found that the abundance of Proteobacteria in the AF and AM groups (which represented the MetS) was significantly higher than that in the NF group (which did not have the MetS), supporting the above theoretical hypothesis. The difference in the abundance of Proteobacteria in NF and NM groups may be due to the influence of gender.

The gender differences in the gut microbiota may determine the gender predisposition to disease (Santos-Marcos et al., 2019a). In the general population with and without MetS, there are many differences between males and females at the genus level. In men with MetS, the gut microbiota is dominated by *Clostridium*, SMB53, *Coprococcus*, *Roseburia*, and *Faecalibacterium*; in women with MetS, the gut microbiota is characterized by the predominance of Cyanobacteria phylum, *Parabacteroides* genus, and the less abundant *Prevotella* genus. In men without MetS, several genera, such as *Clostridium*, *Coprococcus*, Dorea, Lachnospira, *Roseburia*, and *Veillonella*, dominated the gut microbiota. However, the gut microbiota of women without MetS was characterized by several genera, such as *Bacteroides*, Barneciellaceae, *Butyricimonas*, *Parabacteroides*, and Rikenellaceae (Santos-Marcos et al., 2019a).

In contrast, the differences in gut microbiota among the groups in this study did not show such obvious, and there were only significant differences in *Escherichia*/*Shigella*, *Faecalibacterium*, and *Prevotella* between the NF and NM groups. There were no significant differences among genera in both AF and AM groups (Figure 3). Again, as mentioned above, this could be due to the better homogeneity of the samples in this study. A Korean study has demonstrated the influences of different dietary structures and environmental factors on gut microbiota (Noh et al., 2021). Therefore, our study design tried to remove the influence of dietary structure and environmental factors. We selected SCM from the same hospital with an identical dietary structure as the study participants, so there were little differences in the gut microbiota in different sub-groups in this study.

At the genus level, we found that the association between the gut microbiota and the clinical indicators was more complex in all groups. It is thought that some bacteria may significantly affect human metabolism, although their abundance is low (Quigley, 2013). As in the case of *Oscillibacter*, although it was not considered a dominant genus in any groups in this study, it is noteworthy that it was somehow associated with glycemic or lipid indicators in each group. *Oscillibacter* is an essential component of microbial regulation of host metabolism, which affects the release of gut hormones such as PYY and GLP-1 (Olivares et al., 2018; Zeng et al., 2021). PYY, GLP-1, and some other gut hormones regulate glucose and lipid metabolism, fat storage, and appetite in hosts, and atypical antipsychotics, in turn, can regulate these gut hormones. It is hypothesized that as well as regulating PYY and GLP-1, atypical antipsychotics also affect the abundance of *Oscillibacter*, which in turn affects the metabolism of clinical indicators such as blood glucose or lipids in the host (Olivares et al., 2018; Bretler et al., 2019; Martin et al., 2019; Zeng et al., 2021). Therefore, we should pay further attention to *Oscillibacter* in future studies on antipsychotic-mediated nutritional and metabolic disorders.

*Gemmiger* belongs to the phylum Bacillota, which had opposite correlations with the fat distribution of females and males (Min et al., 2019; Zhong et al., 2020), and it also positively correlated with insulin resistance in girls with Idiopathic central precocious puberty (Dong et al., 2020). However, in the present study, we had different findings. In the participants with MetS in this study, *Gemmiger* was positively associated with TG, sdLDL-C, lLDL-C, TC, ApoB, and ApoE in the AF group. In contrast, it showed a negative association with sdLDL-C and TC in the AM group. The finding suggests that there may be a gender difference in the association of *Gemmiger* with Lipid metabolism in MetS of SCM. These new findings of the present study remind us that, on the one hand, *Gemmiger* is extremely closely related to lipid metabolism in the MetA group of SCM. There may be a new mechanism that we do not understand.

On the other hand, we found that although *Gemmiger* was positively associated with six lipid indices in the AF group and negatively associated with two lipid indices in the AM group, in the MA group, which was composed of the AF and AM groups, only sdLDL-C and ApoE were associated with *Gemmiger*. This result was caused by the fact that the MA data were mixed from both AF and AM groups, which obscured the differences related to gender factors. The finding strongly suggests that we must not ignore the importance of gender classification in gut microbiota studies. Suppose we do not consider gender classification but simply mix data from females and males for analysis, once different genders have an opposite influence on the association between a gut microbiota and a clinical index. In that case, the results will lose important information, and the true conclusions will be blurred and diluted.

*Bifidobacterium* had the highest relative abundance in all groups in this study. As an essential probiotic, it is well established that *Bifidobacterium* has positive health benefits for human hosts (O’Callaghan and van Sinderen, 2016). The abundance of Bifidobacteria in gut microbiota is decreased in diabetic patients, and *Bifidobacterium* is negatively correlated with FBG and postprandial blood glucose (Fang et al., 2018). *Bifidobacterium* supplementation can significantly reduce FBG in elderly patients with type 2 diabetes (Moroti et al., 2012), TC, LDL-C, and TG in patients with moderate hypercholesterolemia (Xiao et al., 2003).

However, this characteristic of *Bifidobacterium* was not shown in the present study. In the female participants of this study, only FBG in the AF group was negatively associated with *Bifidobacterium*. What surprised us most was that *Bifidobacterium* lost its probiotic character and showed pathogenic behavior in the NF group of this study. It showed a relatively strong positive correlation with FBG, GA, and LDL-C and a negative correlation with Apo-A1. Since Apo-A1 is the main component of HDL-C, which is responsible for tissue lipid removal and anti-atherosclerosis protection in the human body, it means that the higher the level of *Bifidobacterium* in NF group, the more susceptible to dysglycemia and dyslipidemia in this group.

One reason behind this new finding could be that the Relationship between *Bifidobacterium* and glucose or lipids in SCM is influenced by gender factors, as mentioned before. Another reason could be that the genus *Bifidobacterium* includes many species, and they are not all probiotics. Maybe the superposition of gender factors with clozapine leads to a decrease of some *Bifidobacterium* species with beneficial effects to the host and or an increase of some other *Bifidobacterium* species with un-favorable glycolipid metabolism in the body causing this situation, and it is especially expressed specially and paradoxically in female SCM.

However, our finding differs from the current common belief about *Bifidobacterium* that it is so impressive. We should pay more attention to female SCM to explore the reasons behind this interesting phenomenon. This finding also suggests that the mechanism of metabolic abnormalities caused by clozapine is quite complex. In future in-depth studies, we should pay more attention to the significance of sex differences in gut microbiota for metabolic disorders mediated by clozapine (Mauvais-Jarvis, 2015).

For the first time, We explored the gut microbiota characteristics in SCM with and without MetS and the association between the gut microbiota and clinical glucose and lipid metabolic indices. Although the gut microbiota composition in SCM with and without MetS was essentially the same, there were significant differences in the diversity of their gut microbiota and the abundance of some gut microbiota. We found that at the genus level, the association between gut microbiota and glucose and lipid metabolism indicators was more complex in different gender SCM. On the one hand, the association between particular glycemic or lipid metabolic indicators and gut microbiota may vary among different genders of SCM. On the other hand, the association between a particular gut microbial genus and glucose and lipid metabolism indices may also differ among different genders of SCM. However, since the participants in this study were SCM, their gut microbiota may be affected by gender, disease, medication, and other factors that may complicate our findings. The implication of our findings remains to be determined.

Since the participants in this study were long-term inpatients with schizophrenia from the same hospital, treated with clozapine monotherapy, and having the same daily dietary structure, it was possible to minimize the influence of confounding factors. We tried to ensure that the sample was as homogeneous as possible, which is the most prominent strength of this study.

From a gender perspective, we analyzed the relationship between gut microbiota and glycemic or lipids and observed some new phenomena that may be related to gender factors. It is known that studies in the general population have found that glucose and lipid metabolic processes differ in men and women. Women tend to store adipose tissue in the subcutaneous region, while men tend to deposit visceral fat (Enzi et al., 1986). The prevalence of prediabetes syndrome varies by gender. Impaired fasting glucose is more common in men than women, while impaired glucose tolerance is more common in women (Mauvais-Jarvis, 2015). Women appear more likely than men to develop diseases such as metabolic syndrome and obesity (Kelly et al., 2008). Our new findings support and complement the theories mentioned above, which is another strength of this study.

However, the premise of satisfying sample homogeneity as much as possible led to a relatively insufficient sample size for this study. Therefore, the findings of this study need to be more representative, which is one of the shortcomings of this study. Each gut microbiota may express distinct nutritional and metabolic functions at the species level. Since 16S can only identify at the genus level, we could not identify gut microbiota at the species level. Each species expresses different functions in nutrition and metabolism, so we could not draw precise conclusions about the species, which is another shortcoming of this study. It will be improved by metabolomics and meta-genomic analysis in the future. Also, Sex hormones are associated with gut microbiota sex-dependent differences in humans (Santos-Marcos et al., 2019a). Due to objective constraints, we did not obtain data on the sex hormones of the study participants. No correlation analysis between sex hormones and gut microbiota was performed, which is a regret of this study.

Despite its excellent efficacy, due to the high side effects of clozapine, many psychiatrists waited until the end before having to choose clozapine for schizophrenia (Wimberley et al., 2017). Therefore, schizophrenia patients taking clozapine are generally older. Because schizophrenia most often begins in young adults and continues throughout life, our findings apply only to the age group of this study. They cannot be generalized to all ages.

Finally, although exercise interventions are known to benefit the physical health of patients with MetS, it has been found in recent years that altering the composition of the gut microbiota through dietary interventions can similarly promote nutritional and metabolic abnormalities in MetS patients (Santos-Marcos et al., 2019b), In the future, we can conduct such intervention studies in SCM with metabolic abnormalities.

## Data availability statement

The datasets presented in this study can be found in online repositories. The names of the repository/repositories and accession number(s) can be found below: NCBI–PRJNA991641.

## Ethics statement

The studies involving humans were approved by the Institutional Ethics Committee of Clinical Research Center for Mental Disorders, Shanghai Pudong New Area Mental Health Center, School of Medicine, Tongji University. The studies were conducted in accordance with the local legislation and institutional requirements. The participants provided their written informed consent to participate in this study.

## Author contributions

ZS: conceptualization, methodology, writing—review and editing, and funding acquisition. KD: software, formal analysis, data curation, and visualization. FY and ZS: investigation. XM: resources and supervision. FY: writing—original draft preparation. YZ and SM: project administration. All authors have read and agreed to the published version of the manuscript.
